# Thymoquinone Selectively Induces Hepatocellular Carcinoma Cell Apoptosis in Synergism With Clinical Therapeutics and Dependence of p53 Status

**DOI:** 10.3389/fphar.2020.555283

**Published:** 2020-09-15

**Authors:** Shah Jehan, Chen Zhong, Guangyue Li, Syed Zulqarnain Bakhtiar, Dangdang Li, Guangchao Sui

**Affiliations:** ^1^ Key Laboratory of Saline-alkali Vegetation Ecology Restoration, Ministry of Education, College of Life Science, Northeast Forestry University, Harbin, China; ^2^ State Key Laboratory of Genetic Engineering, School of Life Sciences, Zhongshan Hospital, Fudan University, Shanghai, China

**Keywords:** thymoquinone, hepatocellular carcinoma, cell viability, apoptosis, reactive oxygen species, p53

## Abstract

Thymoquinone (TQ) is a natural compound extracted from the black seeds of *Nigella sativa* Linn. belonging to the Ranunculaceae family. TQ exhibits anti-inflammatory and antineoplastic activities against various cancers. Many therapeutics in hepatocellular carcinoma (HCC) treatments, such as doxorubicin (DOX) and cisplatin (DDP), exhibit considerable side effects on patients. We investigated cytotoxic effects of TQ, alone or in combination with DDP and DOX to HCC cells. TQ exhibited selective killing to HCC HepG2 and SMMC-7721 cells, but relatively low toxicity to normal liver HL-7702 cells. Importantly, when used with DOX or DDP, TQ showed synergistic inhibition of HCC cells, but not HL-7702 cells. We also discovered that Hep3B cells with a p53 null status were more sensitive to TQ than HepG2 and SMMC-7721 cells harboring wild type p53. Consistently, shRNA-mediated p53 silencing in HepG2 cells dramatically enhanced TQ-induced apoptosis, measured by caspase 3 and PARP cleavage. Furthermore, TQ-stimulated increase of reactive oxygen species (ROS) in p53-depleted cells was more pronounced than that in cells with intact p53. In summary, we discovered that TQ synergistically improves the anti-cancer activity of DOX and DDP, and loss of p53 sensitizes HCC cells to TQ-induced apoptosis.

## Introduction

Hepatocellular carcinoma (HCC) is the fifth common malignancy and the third cancer-related cause of death in the world (Elkhoely et al., 2015; Gnutzmann et al., 2018). HCC is a lethal disease accounting for over 5% of human cancers and approximately 80% to 90% of primary liver cancers (Costantini et al., 2013). Only 30% to 40% of HCC patients can be treated with curative approaches, while the majority are subjected to palliative therapies (Llovet et al., 2003). Current HCC treatments include systemic therapy, directed liver therapy, surgical resection, and liver transplantation. The last two options are considered to be useful and curative treatments for liver cancers, but only 15% of the patients are suitable to them because the majority are usually diagnosed at late stages (Roxburgh and Evans, 2008). With major resections and severe cirrhosis, common chemotherapies are not helpful. Therefore, novel chemotherapeutic agents or strategies are urgently needed to treat liver cancer patients with minimal or tolerable side effects (Ashour et al., 2014).

As an anthracycline antibiotic, Doxorubicin (DOX) is a mainstay anti-cancer drug commonly used to treat lymphomas, leukemias, and various solid tumors, including HCC (Park et al., 2012; Kim et al., 2017). The chemotherapeutic application of DOX has been limited due to its considerable side effects on vital organs, including heart, liver, and kidney (Shivakumar et al., 2012). Cisplatin (DDP) is also a well-known anti-cancer drug commonly used for a variety of cancers, including malignancies of bones, soft tissues, blood vessels, and muscles (Dosoize and Madoulet, 2002). DDP also exhibits many adverse effects like other chemotherapeutic agents, including nephrotoxicity, neurotoxicity, and hepatotoxicity (Kursunluoglu et al., 2014).

Thymoquinone (2-methyl-5-isopropyl-1,4-benzoquinone, TQ) is a bioactive compound extracted from the black seeds of *Nigella sativa* Linn. (*N. sativa*) belonging to the Ranunculaceae family and growing in Eastern Africa, the Middle East, and Western Asia (Gali-Muhtasib et al., 2006). The black seeds of *N. sativa* have been used for thousands of years as a food preservative, spice and traditional medicine for numerous diseases (Chopra et al., 1956; Nadkarni, 1976; Yeşilada et al., 1995). Many studies indicate that the black seeds possess activities against various neoplastic diseases, including pancreatic, breast, prostate, skin, renal, colon, cervical and hepatic cancers, as well as myeloblastic leukemia (Salomi et al., 1991; Farah and Begum, 2003; Salim and Fukushima, 2003; El-Mahdy et al., 2005; Khan and Sultana, 2005; Thabrew et al., 2005; Yi et al., 2008; Chehl et al., 2009; Effenberger et al., 2010). Additionally, the extract of the black seeds could enhance the efficacy of generic anti-cancer drugs and also reduced their side effects (Nair et al., 1991; Salomi et al., 1991). As the major constituent of the black seeds of *N. sativa*, TQ has also been demonstrated to have anti-inflammatory activities and antineoplastic effects on various types of cancers (Majdalawieh et al., 2017; Guan et al., 2018). Although the exact mechanism of TQ’s action is not yet known, recent studies indicated that TQ could induce apoptosis *via* elevating reactive oxygen species (ROS) in prostate cancer, primary effusion lymphoma, colon cancer, malignant T-cells, lymphoblastic leukemia, and HL-60 leukemia (Alhosin et al., 2010; El-Najjar et al., 2010; Koka et al., 2010; Effenberger-Neidnicht and Schobert, 2011; Hussain et al., 2011; Dergarabetian et al., 2013). The tumor suppressor p53 has a crucial role in modulating cellular stress conditions and responses to DNA damage and other cytotoxic stresses. Depending on the nature of stimuli and sources of cells, p53 can induce either apoptosis or cell cycle arrest, the latter of which may provide chances to cells for restoring genome integrity under stress conditions (Lane, 1992). For example, SiHa cells expressing high p53 levels showed endurable resistance to hydrogen peroxide-induced apoptosis (Ding et al., 2007).

Recently, the combinatorial use of different anti-cancer agents has become a promising strategy to treat cancers (Zhou et al., 2012). In the current study, we evaluated the anti-cancer activity of TQ alone or in combination with DDP or DOX against HCC and normal liver cells. We discovered selective inhibition of TQ to HCC cells versus normal liver cells, and the synergistic anti-cancer activity of the cotreatment of TQ with DDP or DOX. Additionally, we observed that the rates of TQ-mediated cell death depended on cellular p53 statuses with relatively quick killing of cells lacking functional p53. Our data suggest that TQ is a potential therapeutic agent with decent selectivity and suitable to the treatment of HCC patients harboring mutated or deleted p53.

## Materials and Methods

### Cell Culture and Reagents

The HCC cell lines SMMC-7721 and HepG2, and human normal liver cell line HL-7702 were purchased from the Institute of Biochemistry and Cell Biology (Shanghai, China) and Hep3B was from Shanghai Biowing Applied Biotechnology Co. Ltd. SMMC-7721 and HL-7702 cells were grown in RPMI-1640 medium, and HepG2 and Hep3B cells were grown in DMEM medium, both containing 1% penicillin/streptomycin and 10% fetal bovine serum (FBS) and cultured at 37°C in an atmosphere of 5% CO_2_. TQ (≥ 98%; Cat# 274666), DDP (≥ 99.99%; Cat# P4394), and DOX (≥ 98%; Cat# D1515) were purchased from Sigma-Aldrich (St. Louis, MO). Antibodies against cleaved caspase-3 (Cat# 9664S), caspase-3 (Cat# 9662S), cleaved PARP (Cat# 5625S), and PARP (Cat# 9542S) were purchased from Cell Signaling Technology, Inc. (Danvers, MA). Antibodies for β-actin were purchased from Sigma-Aldrich (Cat# A5441) and GAPDH (Cat# 10R-G109A) was from Fitzgerald Industries International (Acton, MA, USA).

### Cell Viability Assay

Cell viability was determined using the WST-1 assay. Briefly, HepG2, SMMC-7721, Hep3B, and HL-7702 cells were cultured in 96-well plates at a density of 5×10^3^ cells/well in triplicate and cultured overnight. TQ, DDP and DOX were individually dissolved in dimethyl sulfoxide (DMSO) and diluted by corresponding medium. In all treatments, DMSO was used as a vehicle control and the addition of DMSO or chemical agents did not exceed 0.5% (v/v) of culture medium. Cells were treated with different concentrations of TQ, DDP and DOX. For the combinatorial treatments, different concentrations of TQ (30, 40, 50, 60, and 70 μM) were used together with DDP (1.25, 2.5, 5, 10, 20, and 25 μM) or DOX (0.125, 0.25, 0.5, 0.75, 1.0, and 1.5 μM), i.e., TQ+DDP or TQ+DOX. The doses of DDP or DOX were standardized as we previously described (Zhong et al., 2019). After 48 h of treatment, 10 μl of WST-1 solution (Roche, Indianapolis, IN) was added to each well, followed by 3 h incubation at 37°C and measurement on a microplate reader (Molecular Devices, LLC.). The percentage of cell viability in all agents or their combinations at specific concentrations for different time periods was determined by the absorbance at 450 nm normalized by that of the vehicle control. The half-maximal inhibitory concentration (i.e., IC50) values were calculated by using the GraphPad Prism 5.0 software.

### Cotreatment and Determination of Phenotypic Effects

The inhibitory effects of TQ in combination with DDP or DOX were evaluated by the combination index (CI) (Chou et al., 1993) and isobologram (Tallarida, 2001) analyses. For each cell line cotreated by TQ and DDP, an organized system was based on the IC50 value of DDP as a point on the X-axis and the IC50 of TQ as a point on the Y-axis, and a line was drawn between the two points. The datum points of the isobologram corresponding to the IC50 values of TQ and DDP in their cotreatment were plotted in this coordinate system. If the datum point was plotted at the right, near/on the line, or at the left of the line, the inhibitory effect of a cotreatment was determined as “antagonistic”, “additive”, or “synergistic”, respectively. The same method was also used to determine the effect of TQ and DOX cotreatment.

### shRNA Design, Lentiviral Production, Infection, and Ectopic Expression

Small hairpin RNA (shRNA) to target human p53 (sh-p53) was generated as we previously described with a target sequence of GAAACCACTGGATGGAGAATATT (Sui et al., 2002; Stovall et al., 2012) and the sh-p53 expression cassette was subcloned into a lentiviral vector expressing the puromycin resistance gene. Meanwhile, a control shRNA (sh-Cont) with a scrambled target sequence (GGGACTACTCTATTACGTCATT) was constructed in the same way. The ectopic expression of wt p53 was carried out by infecting cells using lentivirus pSL5-p53, which used the β-actin promoter to drive the human wt p53 coding sequence and also expressed the puromycin resistance gene. Lentivirus carrying the empty pSL5 vector was used as a control. Lentivirus production and viral infection followed a procedure that we previously described (Wan et al., 2012).

### Measurement of ROS Levels

Cellular levels of reactive oxygen species (ROS) were measured following previously reported protocols with slight modifications (Dergarabetian et al., 2013; Ashour et al., 2014). The used chemicals in this assay, 2′,7′-Dichlorofluorescein diacetate (DCFH-DA) and N-acetylcysteine (NAC), were purchased from Beyotime Biotechnology (Shanghai, China). When cells were treated by non-fluorescent DCFH-DA, which could be oxidized and converted to fluorescent dichlorofluorescein (DCF) by cellular ROS and thus detected by flow cytometric fluorescence analysis. Briefly, cells differentially treated by chemotherapeutics were washed twice by PBS and then incubated for 20 min in 10 µM of DCFH-DA diluted in DMEM without FBS. The intensity of DCF in the cells was analyzed by flow cytometry (AccuriC6, BD Biosciences, CA).

### Cell Apoptosis Assay

The cell apoptosis assay was conducted as we previously described (Zhong et al., 2019). Cells cultured overnight in 12-well plates were treated by TQ, DDP and DOX or in their combinations, with DMSO as a vehicle control. The treated cells were harvested by trypsinization, washed by ice-cold PBS, and then stained for 10 min by 10 µg/ml of propidium iodide (PI) and 10 µg/ml of Annexin V-FITC provided in an Annexin V-FITC Apoptosis Detection Kit (Cat# A211-02, Vazyme Biotech Co. Ltd, Nanjing, China). The percentage of apoptotic cells was analyzed by a flow cytometer (AccuriC6, BD Biosciences, CA).

### Western Blot Analysis

Cells were treated by each therapeutic alone or in combination, with DMSO as a vehicle control, washed by ice-cold PBS and lysed in a lysate buffer (50 mM Tris pH 7.5, 5 mM EDTA, 0.1% NP40, 300 mM NaCl, and 1× protease inhibitor). The protein concentration of each sample was determined using the Bradford protein assay. The same amount of proteins (20 μg) of each sample was resolved by SDS-PAGE and transferred to poly-vinylidene difluoride transfer (PVDF) membrane, which was then blocked by 5% skim milk in TBST and blotted by a primary antibody, including the antibodies against PARP, cleaved PARP, caspase-3, cleaved caspase-3, p53, β-actin, and GAPDH. After the incubation of a horseradish peroxidase-labeled secondary antibodies, the ECL (Enhanced chemiluminescence) kit (Cat# E411-04, Tanon, Shanghai, China) was used to visualize immunoreactive bands.

### Analyses of Correlation Between p53 Statuses and Clinical Outcomes

Two TCGA datasets of liver cancers (IDs: https://portal.gdc.cancer.gov/projects/TCGA-LIHC and https://portal.gdc.cancer.gov/projects/TCGA-CHOL) and matched clinical outcome information of the patients were downloaded from the TCGA database. In the liver cancer datasets, 398 patients had their p53 statuses and survival data available (Wheeler et al., 2017). Among them, tumor samples from 268 patients were genotyped as wt p53, while the other 130 patients had p53 mutants or deletion. The Kaplan-Meier survival method was used and the graph was generated using the R survival package. The graphs were used to evaluate the correlation between p53 deficient statuses and the overall survival of the liver cancer patients.

### Statistical Analysis

The data represented as the mean ± standard deviation of experiments performed in triplicate. For quantitative analysis, a student t-test and one way ANOVA were performed using the software GraphPad Prism 5.0 (GraphPad, San Diego, CA). A p-value less than 0.05 was considered to be significant *p < 0.05, **p < 0.01, and ***p < 0.001.

## Results

### TQ Exhibited Selective Killing of HCC HepG2 and SMMC-7721 Cells as Compared to Normal Liver HL-7702 Cells

**Figure 1:**
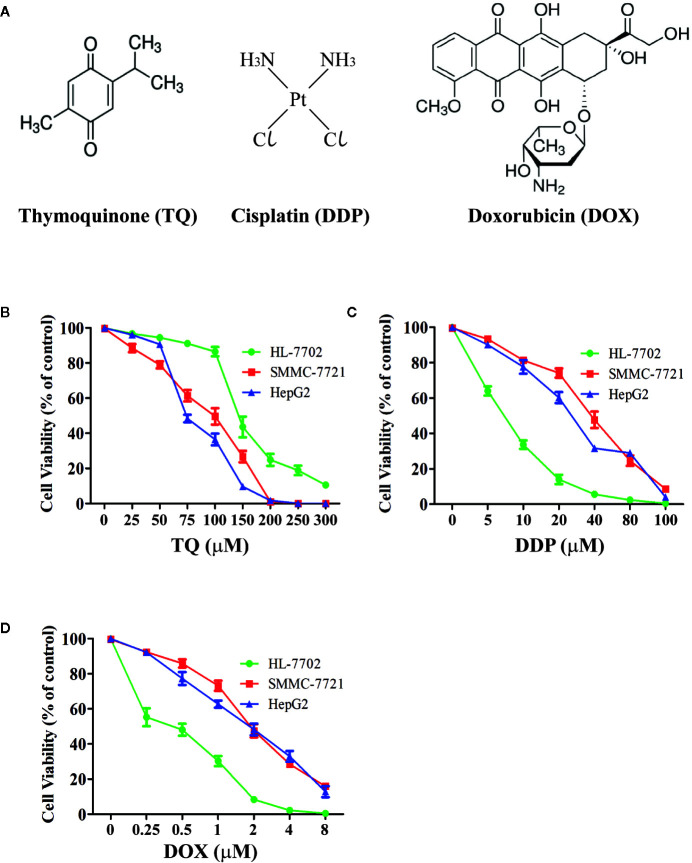
Determining viability of HCC cells and normal liver cells treated by therapeutic agents. **(A)** Chemical structures of therapeutic agents TQ, DDP and DOX. **(B**–**D)** Viability curves of liver cells treated by therapeutic agents. Normal liver cells (HL-7702) and HCC cells (SMMC-7721 and HepG2) were cultured in medium with different concentrations of TQ **(B)**, DDP **(C)**, and DOX **(D)** for 48 h, followed by WST-1 assays to determine cell proliferation. Three independent experiments with each samples in triplicate were conducted with similar results and representative data were presented.

We treated human HCC HepG2 and SMMC-7721 cells, and normal liver HL-7702 cells with different concentrations of TQ, DDP and DOX (**Figure 1A**) for 48 h followed by WST-1 assays to evaluate cell proliferation. All three molecules showed inhibitory effects on cell proliferation of HCC cells in a dose-dependent manner (**Figures 1B–D**). The IC50 values of TQ, DDP and DOX were determined as 84.27 ± 1.72, 29.19 ± 2.94 and 1.88 ± 0.07 μM to HepG2 cells, 91.65 ± 3.26, 39.53 ± 2.52, and 1.66 ± 0.23 μM to SMMC-7721 cells, and 144.35 ± 3.31, 7.02 ± 1.47, and 0.70 ± 0.08 μM to HL-7702 cells, respectively (**Table 1**). The two common anti-cancer therapeutics DDP and DOX, especially the latter one, showed much smaller IC50 values than TQ to all three cell lines, indicating their superior cytotoxic activity over TQ. However, among these three cell lines, DDP and DOX exhibited the smallest IC50 values to HL-7702 cells, but TQ showed an opposite trend with relatively small IC50 values to the two HCC cell lines (**Table 1**). Overall, the data suggested that DDP and DOX had higher toxicity to HL-7702 cells, while TQ showed selective inhibition to the HCC cells.

**Table 1:** The IC50 of TQ, DDP and DOX treatments in HL-7702, SMMC-7721, and HepG2 cells.

Drug Group		IC50 (µM) ± SD		
		HL-7702	SMMC-7721	HepG2
TQ		144.35 ± 3.31	91.65 ± 3.26	84.27 ± 1.72
DDP		7.02 ± 1.47	39.53 ± 2.52	29.19 ± 2.94
DOX		0.70 ± 0.08	1.66 ± 0.23	1.88 ± 0.07
	TQ (μM)			
**DDP**	70	1.75 ± 0.13	4.92 ± 0.30	2.8 ± 0.57
	60	2.93 ± 0.20	11.47 ± 1.57	6.52 ± 1.04
	50	3.83 ± 0.14	16.37 ± 4.11	10.47 ± 1.82
	40	4.58 ± 0.16	19.55 ± 2.63	17.24 ± 1.01
	30	6.10 ± 0.15	24.34 ± 1.73	20.85 ± 1.05
**DOX**	70	0.36 ± 0.02	0.11 ± 0.01	0.17 ± 0.01
	60	0.49 ± 0.04	0.42 ± 0.10	0.41 ± 0.03
	50	0.55 ± 0.01	0.62 ± 0.02	0.68 ± 0.04
	40	0.61 ± 0.06	0.88 ± 0.03	0.98 ± 0.01
	30	0.69 ± 0.03	1.32 ± 0.04	1.26 ± 0.02

The IC50 values of the chemical agents in these cell lines were calculated based on cell viability of three experiments (see **Figure 1A**) using the nonlinear curve-fitting analysis of the GraphPad Prism 5.0 software. The data are presented as the mean ± S.D. of the three independent experiments. The IC50 values of TQ, DDP and DOX showed significantly difference in each of these three cell lines. The IC50 values of each of the three chemicals also showed significantly difference between normal liver cell line (HL-7702) and the HCC cell lines (SMMC-7721 and HepG2).

### TQ in Combination With DDP or DOX Showed Synergistic Inhibition of HCC Cells

To further investigate the effects of TQ in combination with DDP or DOX on HCC cells, we used subtoxic concentrations of DDP and DOX together with TQ to treat HL-7702, SMMC-7721, and HepG2 cells (**Figure S1**). When 70 μM of TQ was used in the cotreatment, DDP and DOX displayed IC50 values of 2.8 ± 0.57 and 0.17 ± 0.01 μM in HepG2 cells, and 4.92 ± 0.30 and 0.11 ± 0.01 μM in SMMC-7721 cells, respectively (**Table 1**), markedly reduced (> 8 folds) in comparison with the IC50 of their individual treatments. When the same concentrations of molecules were used to treat HL-7702 cells, the TQ combined with DDP and DOX displayed IC50 values of 1.75 ± 0.13 and 0.36 ± 0.02 μM, respectively, reduced by less than 2 folds compared to their individual treatments especially in case of DOX. The data suggested that TQ could greatly potentiate the inhibitory activity of DDP and DOX against SMMC-7721 and HepG2 cells, but to a much less extent in HL-7702 cells.

**Figure 2:**
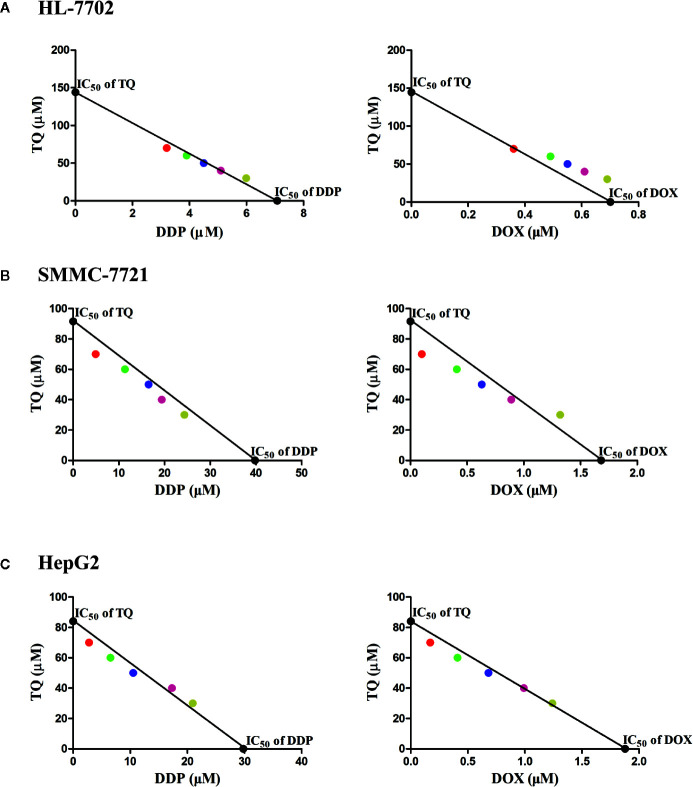
Isobologram analyses of combinatorial cotreatments of liver cells by TQ and DDP or DOX. TQ with a constant concentration of 30, 40, 50, 60 or 70 μM was used together with different concentrations of DDP or DOX in the cotreatments of HL-7702 **(A)**, SMMC-7721 **(B)**, and HepG2 **(C)** cells to determine IC50 values at each TQ concentration. These IC50 data were plotted in the coordinates with IC50 of TQ as the vertical axis and the IC50 of DDP or DOX as the horizontal axis. These circles with specific colors represent a particular concentration of TQ (red: 70 µM, green: 60 µM, blue: 50 µM, pink: 40 µM, yellow: 30 µM).

To assess the potential synergism in the combinatorial use of DDP and DOX with TQ, we carried out isobologram analyses to determine the IC50 values of different cotreatments. In these studies, TQ with a constant concentration of 30, 40, 50, 60 or 70 μM was used with a series of levels of DDP or DOX in the cotreatments to determine IC50 values at each TQ concentration. In HL-7702 cells, most IC50 data of combinatorial treatments stayed on or very close to the lines between the IC50 values of the single drug treatments on the two axes, suggesting that the cotreatment of TQ with either therapeutic likely exerted additive effects on normal liver cells (**Figure 2A**). However, in SMMC-7721 and HepG2 cells, the IC50 data were generally plotted below the lines (**Figures 2B, C**), suggesting that TQ combination with either DDP or DOX was synergistic to the HCC cells. Based on the data of isobologram analyses, we calculated the combination index (CI) values of these cotreatments (**Table 2**). Consistent with the observation from the plots, the TQ cotreatments with DDP and DOX in the HCC cells showed CI values lower than 1.0, indicating synergistic effects of these combinations, while the CI values in HL-7702 cells were close to 1, suggestive of additive inhibitory effects.

**Table 2:** Combination index (CI) values of the cotreatments of TQ with DDP and DOX in HL-7702, SMMC-7721, and HepG2 cells.

Drug group		Combination index (CI)*		
		HL-7702	SMMC-7721	HepG2
	TQ (μM)			
**DDP**	30	1.00	1.06	1.06
	40	0.99	1.02	1.05
	50	0.98	0.95	0.94
	60	0.96	0.93	0.93
	70	0.93	0.88	0.92
**DOX**	30	1.12	1.11	1.02
	40	1.14	0.96	1.00
	50	1.13	0.92	0.95
	60	1.11	0.89	0.93
	70	0.99	0.82	0.92

*The values of CI indicate additive (CI=1), antagonistic (CI>1), or synergistic (CI<1) effects of the inhibition.

### TQ Significantly Boosted DDP- and DOX-Induced Apoptosis

**Figure 3:**
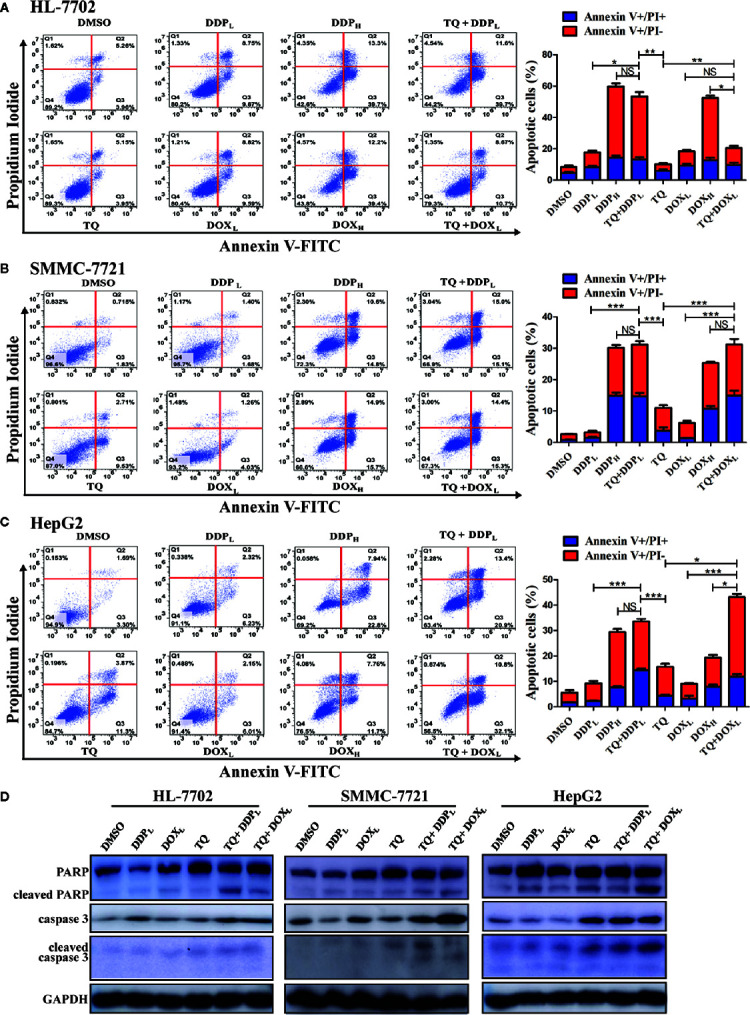
Determining apoptosis of cells treated by therapeutic agents and their combinations. **(A**–**C)** HL-7702 **(A)**, SMMC-7721 **(B)**, and HepG2 **(C)** cells were treated for 48 h by DMSO (0.15%), DOX_L_ (0.25 μM), DDP_L_ (5 μM), TQ (70 μM), DOX_H_ (1.5 μM), TQ+DOX_L_ (70 and 0.25 μM), DDP_H_ (30 μM) and TQ+DDP_L_ (70 and 5 μM), respectively. Then, the cells were stained by Annexin V-FITC and PI, followed by flow cytometry analysis to determine the ratio of apoptotic cells at each treatment condition. Three independent experiments with each samples in triplicate were conducted with similar results and representative data were presented. The data are presented as the mean ± S.D. *p < 0.05, **p < 0.01, ***p < 0.001. NS, not significant. **(D)** Western blot analyses of liver cells treated by therapeutic agents. The cell lysates of the treatments in **(A**–**C)** above were subjected to Western blot analyses using indicated antibodies with GAPDH as a loading control. Representative results from one of the three independent experiments are shown.

Ideal therapeutic treatments would eliminate HCC cells with minimal side effects on normal tissues. For this purpose, we wanted to determine whether TQ could potentiate the anti-cancer activity of DDP and DOX at their subtoxic concentrations. Thus, in the following studies, we used two different concentrations of DDP (5 and 30 μM, designated as DDP_L_ and DDP_H_, respectively) and DOX (0.2 and 1.5 μM, as DOX_L_ and DOX_H_, respectively), with DMSO as a control. HL-7702, SMMC-7721, and HepG2 cells were cultured overnight and then individually treated with DMSO (0.15%), TQ (70 μM), DDP_L_, DDP_H_, TQ+DDP_L_, DOX_L_, DOX_H_, and TQ+DOX_L_. After 48-h treatments, cells were collected and stained with Annexin V-FITC and PI, which detected apoptotic cells at early and late stages, respectively. Cell apoptosis rates were quantified by fluorescence-activated cell sorting (FACS) analysis. Both HepG2 and SMMC-7721 cells treated by TQ+DDP_L_ or TQ+DOX_L_ showed significantly increased apoptosis rates compared to the cells individually treated by DDP_L_ or DOX_L_ (**Figures 3B, C**). Importantly, normal liver HL-7702 cells treated by TQ+DOX_L_ showed an apoptosis rate of 19.33% ± 1.68, very similar to that of 18.85% ± 2.91 by DOX_L_ alone treatment, but significantly lower than that of 50.77% ± 4.09 by DOX_H_ treatment. However, TQ+DDP_L_ treatment of HL-7702 cells caused an apoptosis rate of 53.50% ± 1.47, much higher than that of 18.43% ± 0.89 by DDP_L_ alone, but close to that of 53.08% ± 1.96 by DDP_H_ treatment (**Figure 3A**). The data indicated that TQ could potentiate the proapoptotic effects of DDP and DOX in HCC, and significantly reduced the cytotoxicity of DOX, but to a much lesser extent of DDP, in normal liver cells.

### TQ Enhanced DDP- and DOX-Induced Apoptosis Through Caspase Activation

Western blot analysis was used in evaluating changes of apoptotic markers in liver cells treated by these therapeutic agents. The treatment of HL-7702, HepG2, and SMMC-7721 cells by TQ+DDP_L_ and TQ+DOX_L_ caused a remarkable increase of caspase 3 compared to their individual treatment and the DMSO control (**Figure 3D**). Meanwhile, PARP cleavage was clearly increased in all treatments in HepG2 and SMMC-7721 cells compared to the control; however, in HL-7702 cells, we detected more cleaved PARP by TQ+DDP_L_ treatment than that of TQ+DOX_L_ treatment, suggesting that the latter combination had less cytotoxicity to normal liver cells. Additionally, the cleavage of caspase 3 is generally elevated in HepG2 and SMMC-7721 cells, especially by the TQ+DOX_L_, but showed only a very modest increase in HL-7702 cells (**Figure 3D**). Overall, the Western blot results were generally consistent with the analyses by the flow cytometry showing that the cotreatment of TQ with subtoxic levels of DDP and DOX, especially TQ+DOX_L_, could significantly increase apoptosis of liver cells, but exhibit relatively low toxicity to HL-7702 cells.

### TQ-Mediated Reduction in HCC Cell Viability Depending on p53 Status

**Figure 4:**
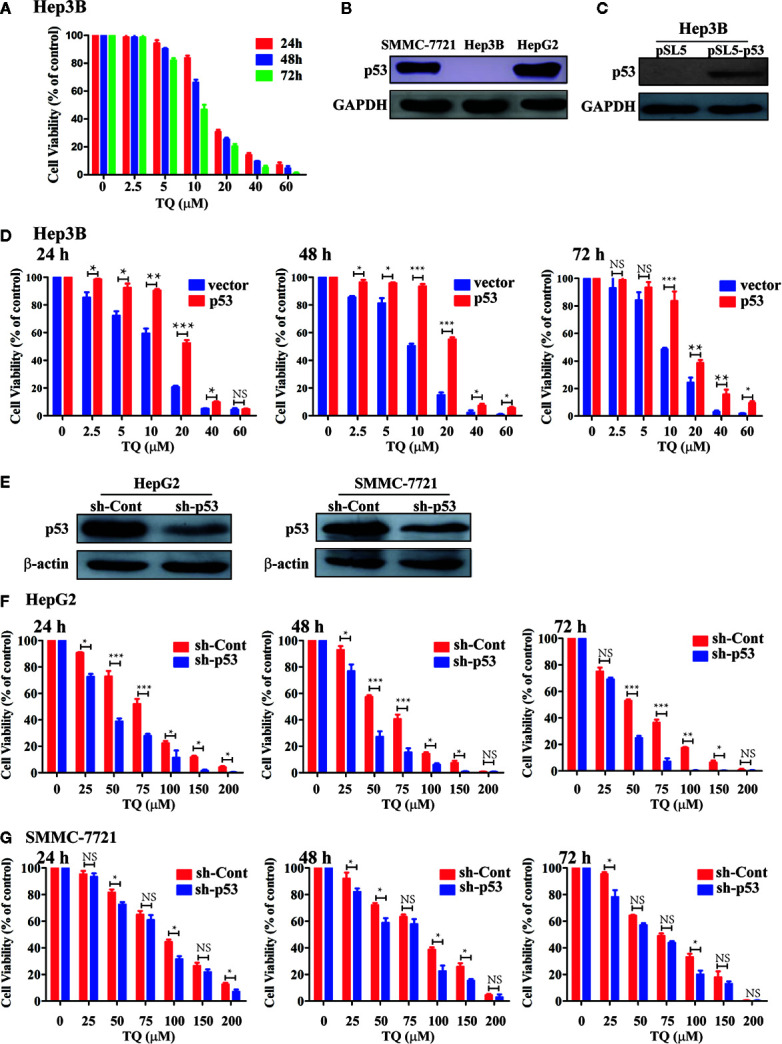
Evaluating the role of p53 in TQ-mediated inhibition of cell viability. **(A, D, F, G)** Cell viability curves of Hep3B cells **(A)**, Hep3B cells with ectopic p53 expression **(D)**, and HepG2 **(F)** and SMMC-7721 **(G)** cells with shRNA-mediated p53 silencing treated by different concentrations of TQ for 24, 48, and 72 h. In these studies, three independent experiments with each samples in triplicate were conducted with similar results and representative data were presented. The data are presented as the mean ± S.D. * p < 0.05, **p < 0.01, ***p < 0.001. NS, not significant. **(B, C, E)** Western blot analyses of endogenous p53 expression in the three HCC cell lines **(B)**, ectopic p53 expression in Hep3B cells **(C)**, and shRNA-mediated p53 knockdown in HepG2 and SMMC-7721 cells **(E)**.

As we obtained the IC50 values of TQ for HepG2 and SMMC-7721 cells, we asked what could determine the response or sensitivity of HCC cells to TQ treatment. As a tumor suppressor and genomic guardian, p53 controls various biological processes and cellular responses to genotoxic or cytotoxic stresses. Thus, we chose liver cancer Hep3B cells with a p53-null background (Bressac et al., 1990) to assess the role of p53 in TQ-mediated inhibition of HCC cell proliferation. Using increasing concentrations of TQ, we could determine the IC50 of TQ in Hep3B cells to be 12.43 ± 2.24 µM after 48 h (**Figure 4A** and **Table 3**), remarkably lower than its IC50 values to HepG2 and SMMC-7721 cells (84.27 ± 1.72 and 91.65 ± 3.26 µM, respectively, **Table 1**) that harbor wild type (wt) p53 (Bressac et al., 1990; Luo et al., 2008). Our Western blot analysis confirmed the presence of p53 in HepG2 and SMMC-7721 cells, and its absence in Hep3B cells (**Figure 4B**). These results suggested that p53 likely played a key role in regulating the sensitivity of HCC cells to TQ treatment. To further prove this prediction, we infected Hep3B cells by lentivirus carrying an empty pSL5 vector or pSL5/p53, with the β-actin promoter driving p53 expression, and confirmed the ectopic p53 expression by Western blot analysis (**Figure 4C**). The cells were then treated by different concentrations of TQ. In three time periods of the TQ treatment, at 24, 48, and 72 h Hep3B cells expressing ectopic p53 generally showed increased viability, compared to the control cells infected by the empty vector (**Figure 4D**). Based on the cell viability curves of these Hep3B cells, we determined the IC50 values of TQ treatment for 24, 48 and 72 h of Hep3B cells expressing wt p53 as 19.41 ± 1.67, 17.54 ± 0.87, and 16.20 ± 1.18 µM, respectively, and the control cells as 13.07 ± 2.81, 11.51 ± 2.24, 16.20 ± 1.18 µM, respectively (**Figure 4D** and **Table 3**). Thus, ectopic p53 expression could indeed reduce the sensitivity of p53-null HCC cells to TQ treatment.

**Table 3:** IC50 ± standard deviation (SD) values of TQ in Hep3B cells carrying the empty vector (pSL5) or expressing ectopic p53 (pSL5-p53).

Hep3B	IC50 (μM) ± SD		
	24 h	48 h	72 h
**pSL5 vector**	13.07 ± 2.81	11.51 ± 2.24	10.57 ± 1.29
**pSL5-p53**	19.41 ± 1.67	17.54 ± 0.87	16.20 ± 1.18

We further evaluated how depletion of endogenous p53 could impact the response of HCC cells to TQ treatment. We produced lentiviruses carrying an shRNA against human p53 (sh-p53) and a scrambled shRNA (sh-Cont) generated as we previously described (Sui et al., 2002). Infection of lentivirus carrying sh-p53 led to reduced protein levels of endogenous p53 in both HepG2 and SMMC-7721 cells (**Figure 4E**). Consistently, compared to the cells expressing sh-Cont, shRNA-mediated p53 knockdown led to significantly elevated sensitivity of HepG2 cells to TQ based on the IC50 values at 24, 48 and 72 h, although this increase was to a lesser extent in SMMC-7721 cells with p53 depletion (**Figures 4F, G** and **Table 4**). Thus, depletion of endogenous p53 could sensitize HCC cells to TQ treatment. Overall, our data strongly suggested that p53 status played a determinant role in TQ-mediated inhibition of HCC cells.

**Table 4:** IC50 ± Standard deviation (SD) values of TQ in HepG2 and SMMC-7721 cells transduced by lentivirus expressing sh-Cont or sh-p53.

Cell line	IC50 (μM) ± SD					
	24 h		48 h		72 h	
	sh-Cont	sh-p53	sh-Cont	sh-p53	sh-Cont	sh-p53
**HepG2**	78.95 ± 4.08	41.05 ± 3.73	68.09 ± 3.33	36.95 ± 3.20	61.94 ± 2.75	35.22 ± 3.35
**SMMC-7721**	89.06 ± 2.97	73.91 ± 4.03	82.52 ± 4.88	78.77 ± 2.12	71.74 ± 3.04	67.96 ± 1.95

### TQ Elevated Reactive Oxygen Species (ROS) to a Pronounced Level in p53-deficient HCC Cells

**Figure 5:**
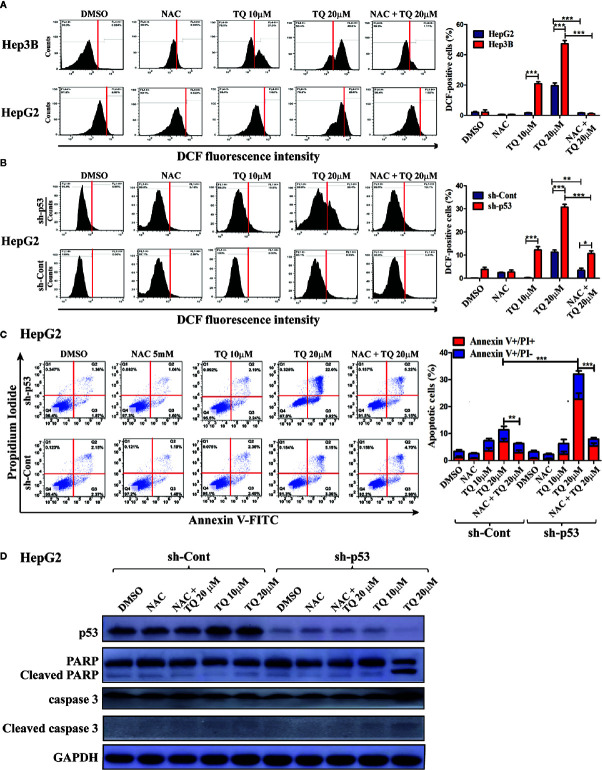
Determining the contribution of ROS induction in HCC cells in response to TQ treatment. A and B. Cellular ROS levels in Hep3B, HepG2 **(A)** and shRNA-expressing HepG2 cells **(B)** after treatment of DMSO (0.15%), NAC (5 mM), TQ (10 and 20 µM), and NAC pretreatment prior to TQ (5 mM and 20 µM, respectively). The flow cytometric graphs are shown at the left and the quantitative bar graphs are shown at the right. **(C, D)** Flow cytometric analyses of apoptotic cells stained by Annexin V-FLTC and PI **(C)**, and Western blot analyses of apoptosis protein markers **(D)** of HepG2 cells treated in the same design of **(A, B)**. The quantitative bar graphs of apoptotic HepG2 cells for **(C)** are shown at the right. In these studies, three independent experiments with each samples in triplicate were conducted with similar results and representative data were presented. The data are presented as the mean ± S.D. * p < 0.05, **p < 0.01, ***p < 0.001.

We further explored the mechanism underlying increased sensitivity of p53-deficient HCC cells to TQ. Reactive oxygen species (ROS) play a key role in promoting cell apoptosis (Redza-Dutordoir and Averill-Bates, 2016). Thus, we asked whether TQ could differentially trigger ROS production in HCC cells with different p53 status. HepG2 cells harboring wt p53 and Hep3B cells with a p53-null status were pretreated with 5 mM N-acetylcysteine (NAC**), a** ROS scavenger, for 2 h, followed by TQ treatment for 6 h. The ROS positive cells after the treatment were stained by DCF-DA, a fluorescent ROS-detecting dye, and the ROS levels were quantified by flow cytometry. As shown in **Figure 5A**, Hep3B cells exhibited a marked increase of ROS levels when treated by 10 and 20 µM of TQ, which was largely diminished by the pretreatment of NAC. However, HepG2 cells showed much reduced ROS elevation, especially at 10 µM of TQ (**Figure 5A**). To interrogate whether p53 played a role in decreasing ROS production, we repeated the experiment with HepG2 cells carrying sh-Cont or sh-p53. As shown in **Figure 5B**, p53 knockdown significantly increased cellular levels of ROS when treated by TQ, even in the cells pretreated by NAC. The data strongly indicated that TQ treatment could promote the production of ROS in a p53-dependent manner, which may contribute to cell apoptosis.

### Depletion of p53 Sensitizes HepG2 Cells to TQ-Induced Apoptosis

As we observed p53 knockdown could reduce cell viability caused by TQ-treatment compared to cells with intact p53, we asked whether this was due to enhanced cell apoptosis. Thus, with or without pretreatment of 5 mM NAC for 2 h, the HepG2 cells harboring sh-p53 or sh-Cont were cultured in medium containing 10 and 20 µM of TQ for another 24 h, followed by staining of Annexin V-FITC and PI, and analyzed using a flow cytometer. The apoptotic rates of HepG2 with sh-p53 at 10 and 20 µM were determined as 8.43% ± 0.72 and 33.16% ± 4.33, respectively, significantly higher than those of cells harboring sh-Cont (4.56% ± 1.06 and 16.73% ± 3.19, respectively, **Figure 5C**). Importantly, the apoptotic rates of cells with either sh-p53 or sh-Cont were generally reduced when cells were pretreated by 5 mM of NAC followed by 20 µM of TQ. We further evaluated changes of apoptotic markers in these cells by Western blot analyses. As shown in **Figure 5D**, HepG2 cells with shRNA-mediated p53 silencing showed increased cleavage of PARP and caspase 3 in response to TQ treatment, compared to the sh-p53 cells treated by DMSO and the cells expressing sh-Cont. The data suggested that p53 deficiency could sensitize HCC cells to TQ treatment through enhancing cell apoptosis.

### p53 Deficiency Negatively Correlates With the Survival of Liver Cancer Patients

**Figure 6:**
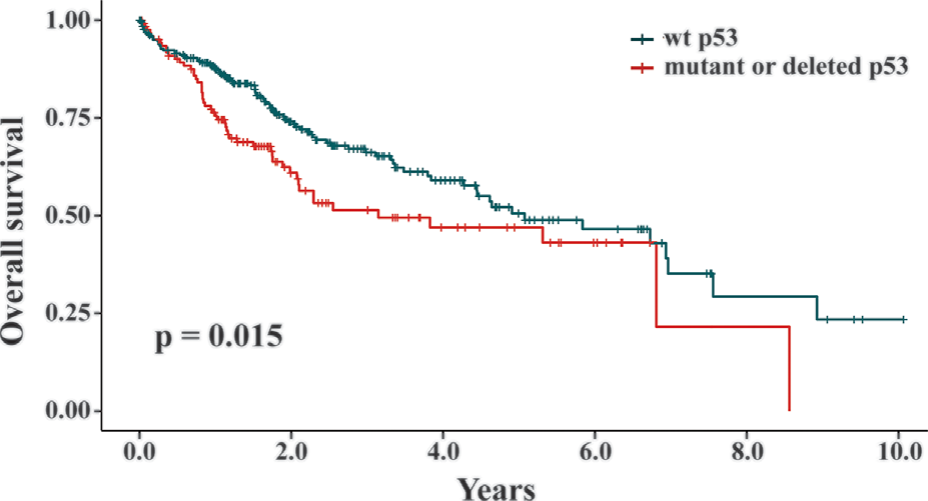
The correlation between p53 statuses and overall survival of liver cancer patients. In the TCGA datasets TCGA-LIHC and TCGA-CHOL, 398 liver cancer patients with their p53 statuses and survival data available were analyzed by the Kaplan-Meier survival curves. Among them, 268 patients were genotyped as wt p53 and the other 130 patients were p53 mutants or deletion. The p value of 0.015 indicated that the liver cancer patients with a wt p53 status showed statistically better survival rates than the patients with mutant or deleted p53.

Our data revealed that the presence of wt p53 reduced the response of HCC cells to TQ-induced cell growth retardation. As a key suppressor of oncogenic transformation, p53 is frequently mutated or deleted in various malignancies, including liver cancer (Hollstein et al., 1991; Jeng et al., 2000; Rivlin et al., 2011). Thus, increased sensitivity of HCC cells with a p53 null status suggested that TQ could be a more potent anti-cancer agent in HCC cells or tumors with p53 deficiency than to those carrying functional endogenous p53. Generally, p53 alteration can initiate the oncogenic process or promote cancer progression. To assess whether p53 statuses are truly involved in liver cancer development, we analyzed the TCGA datasets TCGA-LIHC and TCGA-CHOL, and found 398 liver cancer patients with their p53 statuses and survival data available (Wheeler et al., 2017). Among them, liver cancers from 268 patients were genotyped as wt p53, while the tumors from the other 130 patients were p53 mutants or deletion. We utilized the Kaplan-Meier survival curves to analyze these patients and discovered that p53 deficient statuses significantly correlated with short overall survival of liver cancer patients compared to those with wt p53 (p = 0.015, **Figure 6**). Thus, the relatively high potency of TQ to p53-null HCC cells suggested that TQ-based therapies would be more efficient or suitable to liver cancer patients with p53 alterations that generally have poor clinical outcomes, which highlights the clinical significance of the current study.

## Discussion

HCC is the fifth most common malignancy and the third leading cause of cancer-related death globally (Parkin, 2006). About 80% to 90% of patients with primary liver cancers are diagnosed as HCC and they usually die of the disease within 6 to 20 months. Regular therapeutic strategies are generally ineffective in HCC patients with late diagnosis, cirrhosis or major resection (El-Serag et al., 2003; Llovet et al., 2003; Ashour et al., 2014). Patients with late stages of HCC are typically treated by high doses of antineoplastic drugs that can often induce severe adverse effects in vital organs, such as the heart and kidneys (He et al., 2016). Therefore, it is urgently needed to develop novel and effective therapeutic strategies to block HCC progression or cure this deadly disease with minimal side effects.

As a natural compound, TQ has been demonstrated to possess anti-inflammatory and antineoplastic activities in various types of cancers, including liver cancer (Elkhoely et al., 2015; Majdalawieh et al., 2017; Guan et al., 2018); however, its potential side effects on normal liver has not been reported. Our current study indicated that TQ had much reduced toxicity to normal liver cells compared to that seen in different HCC cell lines, although its potency in killing these liver cancer cells was lower than that of DDP and DOX. These results implicated great tumor-killing selectivity of TQ in its future clinical applications. Notably, previous reports indicated that pretreatment of TQ exerted protective effects against cardiotoxicity caused by doxorubicin and cisplatin applications in mouse models (Badary et al., 1997; Al-Shabanah et al., 1998). Accordingly, we investigated whether TQ combination with subtoxic levels of DDP or DOX could show high antiproliferative effects on HCC cells but low toxicity to normal liver cells. As a result, we observed that TQ could synergistically improve tumor suppressive activities of DDP and DOX against HepG2 and SMMC-7721 cells, while the combinatorial treatments, especially TQ and DOX, showed low toxicity to normal liver HL-7702 cells. Consistent with our results, a recent study demonstrated that TQ could significantly potentiate the anti-cancer activity of cyclophosphamide against breast cancer cells (Khan et al., 2019). Furthermore, we found that the cotreatment could induce more pronounced apoptosis of HCC cells than that of normal liver cells, suggesting that TQ exerted its cancer cell-inhibitory and normal cell-protective activities in these studies.

The tumor suppressor p53 plays a crucial role at cellular stress conditions and in responses to DNA damage. Depending on the nature of stimuli and genetic backgrounds, p53 can induce either apoptosis or cell cycle arrest, the latter of which grants the cell the time and chance of repairing DNA or other cellular damages caused by stresses (Lane, 1992). Thus, we investigated the effects of p53 status on HCC cell response to TQ treatment. We discovered that Hep3B cells without endogenous p53 expression showed remarkably higher sensitivity than HepG2 and SMMC-7721 cells harboring wt p53. In addition, p53 silencing in HepG2 cells or its ectopic expression in Hep3B cells could increase or reduce cell sensitivity to TQ, respectively. The underlying mechanism of this phenomenon could be that p53 activation in HCC cells with wt p53 may cause cell cycle arrest to give cells time to repair damage caused by TQ. However, in cells without functional p53, this mechanism is deficient, thus leading to quick cell death. A study using osteosarcoma cells also indicated that MG63 cells with p53-null status were more sensitive to TQ than normal osteoblast and MNNG cells expressing p53 (Roepke et al., 2007). It is noteworthy that the increased sensitivity of p53 knockdown in SMMC-7721 cells was reduced compared to HepG2 cells. Different genetic backgrounds between the two cell lines could contribute to this variance, which needs to be investigated in future studies. As an important tumor suppressor, p53 and its regulated pathways are commonly compromised in various cancer cells through different mechanisms. It is possible that TQ can only restore p53 activity in certain scenarios of cancer cells, but not the others, which may determine the response of cancer cells to TQ treatment.

A previous study indicated that TQ could increase ROS levels and induce apoptosis of HCC cells (Ashour et al., 2014). Another study indicated that inhibition of p53 elevated intracellular ROS levels in cervical carcinoma SiHa cells (Ding et al., 2007). Consistently, our current study also demonstrated that TQ treatment caused highly increased ROS in Hep3B cells compared to that in HepG2 cells, while HepG2 cells transduced by sh-p53 lentivirus showed more pronounced ROS elevation than their sh-Cont counterparts. Additionally, we also used Western blot analyses of different apoptotic markers to demonstrate relatively increased apoptosis in TQ-treated p53-null or -deficient cells compared to p53-intact cells. As a well-characterized tumor suppressor, p53 has been demonstrated to modulate numerous processes related to tumorigenesis, including DNA damage repair, cell cycle arrest, apoptosis, senescence, etc. Deficiency of p53 can lead to malignant transformation to initiate tumors; meanwhile, its mutation or deletion may also occur at late stages of cancers, which can augment cancer progress and drug resistance (Zhou et al., 2018). Consistently, our analysis of the TCGA data and previous studies demonstrated that p53 mutations and deletion were associated with cancer invasiveness and poor outcome of HCC patients (Jeng et al., 2000; Liu et al., 2012; Zhan et al., 2013). Thus, the activity of TQ in selectively killing HCC cells with modest adverse effects to normal cells suggests its advantage in clinical applications for liver cancer patients with p53 deficiency.

## Conclusion

Our study revealed that TQ significantly boosted the antineoplastic activities of DDP and DOX. These combinatorial treatments strongly induced apoptosis of HCC cells by caspase activation, but exhibited relatively low cytotoxicity to normal liver cells compared to that observed with conventional therapeutics alone. Thus, TQ in combination with a subtoxic level of clinical therapeutics, especially DOX, represents a promising and safe strategy in liver cancer therapies, which can efficiently kill the tumor cells and minimize the adverse effects generated when the therapeutics is used in high dosage. Importantly, we also discovered that lack of p53 could sensitize HCC cells to TQ-caused cell apoptosis, suggesting that TQ can be potentially used to treat cancer patients with deficient p53. Overall, our data demonstrated that TQ, as a natural compound, could selectively inhibit HCC cell proliferation and its combinatorial use with cancer therapeutics is of important clinical significance.

## Data Availability Statement

The raw data supporting the conclusions of this article will be made available by the authors, without undue reservation, to any qualified researcher.

## Author Contributions

SJ, DL, and GS conceived the project, wrote the manuscript and generated the figures. SJ, CZ, and SZ conducted experiments. GL performed bioinformatic analyses. All authors contributed to the article and approved the submitted version.

## Funding

This work was supported by the Fundamental Research Funds for the Central Universities (2572020DY13) to GS, the National Natural Science Foundation of China (81672795 and 81872293) to GS.

## Conflict of Interest

The authors declare that the research was conducted in the absence of any commercial or financial relationships that could be construed as a potential conflict of interest.

## Abbreviations

DCFH-DA, 2’,7’-dichlorodihydrofluorescein diacetate; DDP, cisplatin; DMSO, dimethyl sulfoxide; DOX, doxorubicin hydrochloride; HCC, hepatocellular carcinoma; NAC, N-acetylcysteine; PI, propidium iodide; ROS, reactive oxygen species; TQ, thymoquinone.
