# Supplementary material for: Thymoquinone Selectively Induces Hepatocellular Carcinoma Cell Apoptosis in Synergism With Clinical Therapeutics and Dependence of p53 Status
Source: Front Pharmacol. 2020 Sep 15;11:555283. doi: 10.3389/fphar.2020.555283 (PMC7522566; doi:10.3389/fphar.2020.555283)
Supplement: Supplementary file 1 [file Image_1.pdf]

Thymoquinone selectively induces hepatocellular carcinoma cell apoptosis in synergism with clinical therapeutics and dependence of p53 status

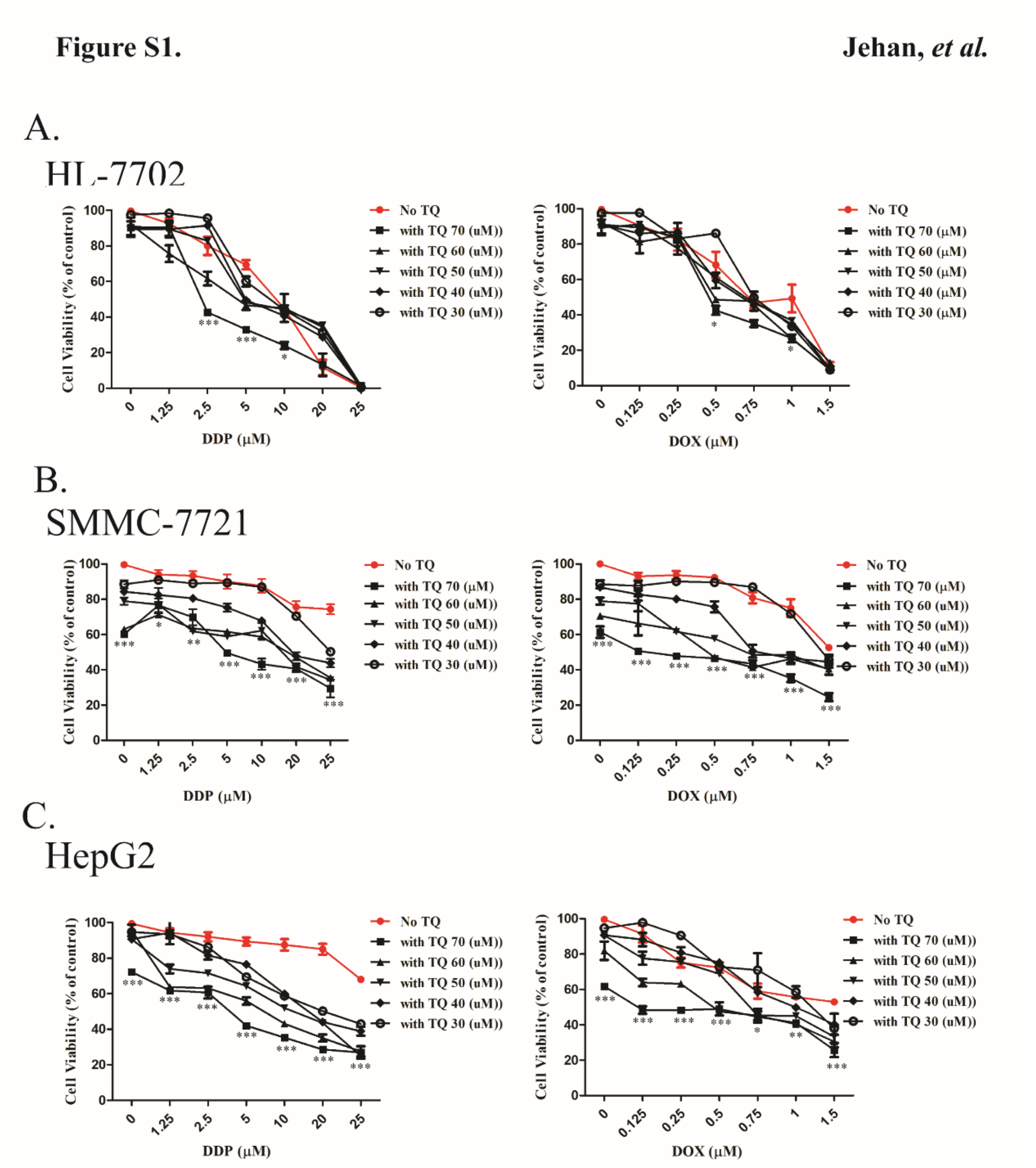

Figure S1. Thymoquinone (TQ) potentiates the antineoplastic activities of cisplatin (DDP) and Doxorubicin (DOX) in HCC cells and normal liver cells. HepG2 (A), SMMC-7721 (B) and normal liver HL-7702 (C) cells were treated DDP, DOX and their combinations with TQ for 48h and followed by WST-1 assay to determine cell viability. IC50 values were calculated with nonlinear curve-fitting analysis with the GraphPad Prism 5.0 software. Many pairs of data between "No TQ" and "with TQ" showed a significant difference. However, due to the limited space, we only indicated the significant difference between the treatments of "No TQ" and "with TQ 70 μM". The data are presented as the mean ± S.D. * $p < 0.05$, **$p < 0.01$, ***$p < 0.001$.
